# Mammograms and breast arterial calcifications: looking beyond breast cancer: a preliminary report

**DOI:** 10.1186/1756-0500-4-207

**Published:** 2011-06-20

**Authors:** Rachael A Akinola, Okeoghene A Ogbera, Josephine AA Onakoya, Chris E Enabulele, Idowu O Fadeyibi

**Affiliations:** 1 Department of Radiology, College of Medicine/Lagos State University Teaching Hospital (LASUTH), Ikeja- Lagos, Nigeria; 2 Department of Medicine, Endocrine Unit, College of Medicine/Lagos State University Teaching Hospital, (LASUTH), Ikeja-Lagos, Nigeria; 3 Department of Chemical Pathology, College of Medicine/Lagos State University Teaching Hospital (LASUTH), Ikeja- Lagos, Nigeria; 4 Department of Community Health and Primary Health Care, College of Medicine/Lagos State University Teaching Hospital (LASUTH), Ikeja-Lagos, Nigeria; 5 Department of Surgery, Burns/Plastic Surgery Unit, Lagos State College of Medicine/Lagos State University Teaching Hospital (LASUTH), Ikeja-Lagos, Nigeria

## Abstract

**Background:**

To find out the prevalence, clinical and biochemical correlates of Breast Artery Calcification (BAC) in the Nigerian women.

**Findings:**

This is a cross sectional study involving 54 consecutive adult female subjects sent to the Radiology Department of the Lagos State University Teaching Hospital (LASUTH), Ikeja-Lagos, Nigeria for screening and diagnostic mammography. The study was carried out for a period of five months.

The prevalence of BAC was 20%. Ageing was found to be related to BAC. Cardiovascular risk factors including diabetes mellitus (DM), hypertension, obesity, alcohol ingestion, use of oral contraceptives and hormone replacement therapy, were not significantly related to the presence of BAC in this study.

**Conclusion:**

This study showed that though the presence of BAC in a mammogram is related to age, it may not predict or serve as a significant marker for cardiovascular diseases (CVD) in women in our environment.

## Background

Arterial calcification is a common feature of atherosclerosis which can be elicited with conventional radiological imaging as calcium deposits in the arterial wall [1]. The appearance of calcium in different vascular beds occurs 10-15 years later in women than in men [1]. Breast Artery Calcification (BAC) on mammography was seen in 9% of women in a study that was carried out in Utrecht, The Netherlands. BAC has been identified as calcific medial sclerosis of medium sized breast arteries and was reported to be associated with cardiovascular risk factors including diabetes mellitus [DM], hypertension [2], coronary artery disease (CAD) and cardiovascular mortality [1,3-6].

Histopathologically, medial calcification differs from intimal calcification in the absence of signs of inflammation and lipid deposits [1,7]. Conventional x-ray techniques cannot however differentiate between intimal and medial calcification. Medial calcification is finer and diffuse in smaller vessels, while intimal thickening in large and medium sized arteries, are large and discontinuous [1,3,7]. Atherosclerosis and medial sclerosis appear more frequently and at an earlier age in diabetic patients [8]. Fiuza et al [9] suggested that mammographic finding of BAC calls for more attention from the Radiologist and should not be excluded from reports.

Vascular calcifications in the breast are defined as the presence of parallel linear calcified deposits along the course of a vessel that is seen on at least one view of a mammogram [7,10]. They are called Monckeberg calcifications and involve the middle layer of arteries [3,7,10]. They are diffuse, thin and involve the whole circumference of the peripheral arteries making the vessels stiff. These are uncommon in patients less than 50 years old and are found in about 9.1% mammograms [2,3,7,10,11]. The prevalence of vascular calcification ranges from 9-17% and increases with age, exceeding 50% in women aged 65 years and above [10,12]. Other studies have found associations between BAC and chronic diseases including kidney failure, autonomic neuropathy and hypervitaminosis D [10] and also with Cardiovascular risk factors which include increasing age, parity, obesity, cigarette smoking, alcohol ingestion, use of oral contraceptives or hormone replacement therapy, diabetes, hypertension and deranged lipid profile [2].

BAC that is seen in breast cancer screening mammograms may also be associated with disorders related to increased or accelerated atherosclerosis [13]. Increased parity has been associated with decrease in breast cancer risk but increases the occurrence of BAC [13]. Breast cancer screening may also aid early detection of enhanced CVD risk among otherwise healthy women [13].

This study is aimed at finding the prevalence of BAC and its relationship with cardiovascular (CVD) risk factors in Lagos, Nigeria.

## Methods

Fifty-four consecutive adult female subjects that were sent for screening and diagnostic mammography between February and June 2010 were recruited for this prospective study. All the subjects consented to the study. Interviewer administered questionnaire which included information on the biodata and anthropometric measurements comprising of waist circumference, height and body weight were answered by the patients. The Body Mass Index (BMI) was calculated using the formula- weight/height^**2 **^(kg/m^2^) for the patients [10]. The waist circumference was measured at the midpoint between the inferior margin of the 10th rib and the crest of the ilium with a tape measure. The medical history of any previous stroke and or heart failure and necessary information on the risk factors for CVD were also obtained.

The mammograms, cephalocaudal (CC) and mediolateral oblique (MLO) views were done using a Villa Systemi Stereotactic Mammography machine and these were assessed for BAC using a well illuminated viewing box and hand lens.

An excel data spreadsheet was used to record all these information. Statistical Package for Social Sciences (SPSS), version 17, Chicago Illinois was used for statistical analysis

### Statistical Analyses

Descriptive results for continuous variables were expressed as mean ± standard deviation (SD). Biochemical and clinical parameters were compared between the study subjects with and without BAC using independent samples t-test for continuous variables and chi-square for categorical data. Possible predictors for the presence of BAC were evaluated using binary logistic regression. The variables entered in the model included CVA risk factors (obesity, central obesity, elevated LDL, elevated total cholesterol and dyslipidaemia). A p-value < 0.05 was considered statistically significant.

### Biochemical analyses

#### Lipid parameters

Fasting venous blood samples were taken for the determination of High Density Lipoprotein-Cholesterol (HDL-C), and Triglyceride (TG). HDL-C was determined by the precipitation method [14] and TG estimated using a standardized kit employing enzymatic hydrolysis of TG with lipases [15]. Fasting Blood Sugar (FBS) level estimation was done as a point of care test using a glucometer (Glucolab manufactured by Infopia Limited, Korea Republic) from fasting capillary blood samples. Total cholesterol (TCHOL), Low density Lipoprotein Cholesterol (LDL-C), Very Low Density Lipoprotein (VLDL) and electrolyte and urea (E&U) were also assessed. The serum levels of Total protein and albumin were also checked.

### Operational definitions

**Figure 1:**
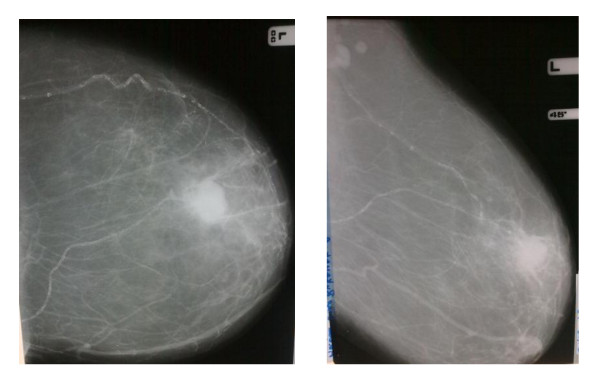
**Showing the CC and MLO views of a mammogram with "Tram-like calcifications" in the upper quadrant of the breast in an 80 year old woman who incidentally also had a malignant breast mass**.

1. Abnormal lipid parameters refers to elevated TCHOL, LDL, TG and reduced HDL [16].

2. Dyslipidaemia refers to serum triglycerides (TG) level of at least 150 mg/dl, high-density lipoprotein cholesterol (HDL-C) level of less than 50 mg/dl [17].

3. Hypercholesterolaemia and elevated LDL refers to TCHOL > 200 mg% and LDL-C > 100 mg% [16].

4. Obesity was defined as abnormal or excessive fat accumulation that may impair health. Obesity refers to a BMI >to 30 [18].

5. Central obesity was when the waist circumference (WC) dimension was greater than 88 cm [17].

6. BAC was diagnosed when easily recognized characteristic pattern of two linear parallel calcific lines giving a "railroad track" configuration described by Sickles was seen on the mammogram [3,7,10,19], (Figure 1).

## Results

This study revealed that the older the patient, the more the possibility of developing BAC, as shown in (Table 1). Most of the BAC seen in this study occurred between 60 - 69 years while none was seen below 40 years, (Table 1).

**Table 1:** Distribution of BAC according to age decades in the study subjects

Age Range(years)	Subject Fequency (%)	BAC Frequency (%)	BAC Frequency/Subject frequency (%)
**30 - 39**	1 (1.9)	0 (0)	0%
**40 - 49 years**	20 (37.0)	2 (18.2)	10%
**50 - 59 years**	21 (38.9)	2 (18.2)	9.5%
**60 - 69 years**	10 (18.5)	5 (45.5)	50%
**> 70 years**	2 (3.8)	2 (18.2)	100%

Only six (11.15%) participants had a significant history of alcohol ingestion, none of them smoked cigarette and none had a past or present history of cardiovascular accident (CVA) or heart failure. The potential risk factors for BAC in the study subjects are shown in (Table 2).

**Table 2:** Distribution of some risk/potential factors for BAC

Parameter	No. (Frequency)%
Breast Feeding	48 (89%)
Hypertension	19 (35%)
DM	5 (9%)
History of usage of oral contraceptive	10 (18%)
History of use of hormone replacement therapy	4 (8%)
Alcohol ingestion	6 (11,1%)
Menopausal status	34 (62%)

About one fifth of the study subjects had BAC, with a prevalence of 20% and of all the correlates, only the difference in the ages of subjects with and without BAC was statistically significant (p = 0.002),(Table 3). Those that had BAC were obese with an average BMI of 31.3 Kg/m^2 ^though not statistically significantly different from those that did not have BAC. Their fasting blood sugar was however within normal limits. All the clinical and biochemical correlates were not statistically significant, (Table 3).

**Table 3:** Comparison of clinical and biochemical variables between the subjects with and without BAC

Variables	Subjects with BAC	Subjects without BAC	p value
Age (years)	60 (10.1)	50.9 (7.8)	0.002
BMI (Kg/m^2^)	31.3 (4.9)	28 (4.9)	0.09
WC (cm)	93.5 (10.6)	93.4 (17.5)	0.9
Weight (Kg)	78.9 (11)	73.7 (13.5)	0.2
T chol (mg%)	177.4 (32.9)	178.4 (36.9)	0.1
TG (mg%)	79 (28.2)	65.7 (22.5)	0.3
HDL-C (mg%)	50 (9)	45.7 (14.3)	0.6
LDL-C (mg%)	126 (37.9)	120.6 (32)	0.9
Total protein	7.1 (0.3)	7.1 (0.6)	0.9
FBS	85 (13.9)	86.1 (15)	0.8
Albumin	3.5 (0.3)	3.7 (0.6)	0.08

*Results are expressed as means and standard deviations

Table 3 also shows that although the mean age of the subjects with BAC was significantly higher than the subjects without BAC, all other mean values of their clinical and biochemical parameters were comparable.

**Figure 2:**
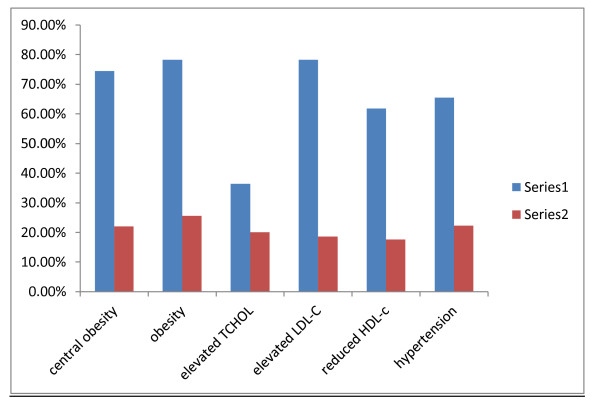
**Distribution of cardiovascular risk factors in the study subjects and in those with vascular calcifications**. **Series 1**: Proportion of study subjects with CVD abnormalities **Series 2: **Proportion of the subjects with stated CVD abnormalities that had mammary vascular calcification.

The distribution of CVD risk factors were similar in subjects with and without BAC and Figure 2 shows that apart from the elevated total cholesterol, the proportion of subjects with BAC who had the CVD risk factors depicted in the figure was less than 50%.

Of the 34 subjects who were menopausal, only 3 (8.8%) were found to have vascular calcifications, and the difference in the occurrence of BAC in subjects with or without history of the use of oral contraceptives was not statistically significant (p = 0.09). Only One subject among those with history of the use of HRT had vascular calcification and there was no statistically significant difference in the occurrence of vascular calcifications among the subjects with and without history of usage of HRT (p = 0.7). Vascular calcification was not found in any of the participants with a history of alcohol ingestion or DM, while all except 2 (18,2%) of the subjects with BAC breast fed. This study has therefore shown that none of the possible predictors of BAC that were investigated was statistically significant, (Table 4).

**Table 4:** Possible Predictors of Vascular calcification

Parameter	Odds ratio	95% CI	p value
Central obesity	0.7	0.1-5.9	0.8
Obesity	0.9	0.01-2.5	0.9
Hypercholestolaemia	1.2	0.26-6.4	0.7
Reduced HDL	0.8	0.2-3.7	0.8
Elevated LDL	0.3	0.05-2.5	0.3

### Sites of BAC

BAC was found in 5(45.5%) breasts on the right and 2(18.2%) on the left. It occurred bilaterally in 4(36.4%). Two (18.2%) of those that had BAC were nulliparous. All the quadrants of the breasts were equally affected (upper, 36.4%, lower, 36.4% and upper and lower, 27.2%).

## Discussion

The significance of BAC on mammograms is still debatable. While some authors claim that it might be a useful means of predicting CVD in women, others are of contrary opinion [1,8,12,19].

In the present study, increasing age was found to be associated with BAC. Women with BAC were significantly older than those without BAC in the present study. Maas et al [1] noted in their study that BAC was associated with increasing age, pregnancy and lactation but not with the traditional CVD risk factors. Kim et al [7] claim that Sickle and Galvin and Taskin et al [20] also found that at mammography, visible BAC are positively correlated with increased age. BACs were seen more frequently in postmenopausal women in other studies [7], while a study in Brazil by Ferreira et al [21] found that significantly more women with BAC had CVD compared to women who did not have BAC. This is however contrary to the finding in this study where only 3 (8.8%) of the postmenopausal women had BAC.

Parity and breast feeding did not show any significant relationship with BAC in the present study. Previous studies have not been conclusive as to whether pregnancy and lactation may have any role in the calcification of other vascular beds besides breast arteries [1].

Vascular calcifications in the breast usually have a lipid component and resemble calcifications seen in other arteries [10]. The mechanism of deposition is still unknown. Oliveira et al [10] claim that when present, BAC was always bilateral. This may be a reflection of the atherosclerotic process and consequent vascular calcification affecting the whole arterial system. This was not true in this study as only 36.4% had bilateral BAC.

Although BAC was reported by Baum et al [8] as a sign of coexisting diabetes, none of the diabetics in this study had BAC. Findings in this study were also contrary to the study by Moshyedi et al [19], who reported that nearly all women in their study group younger than 59 years with BAC also had CAD and DM [7,11].

Studies have listed age, hypertension, hypercholesteraemia, DM and menopause as the risk factors for CVD [22] in BAC-positive populations. Age is the only significant factor that was found in the present study. This is similar to the findings by Kataoka et al [12].

The prevalence of BAC in this study is much higher than those of previous studies which were mostly done among the Caucasians [10,12,13,22]. The reason for the high prevalence in our environment is not known. Factors that may arise from racial differences between the subjects of this study (who are mostly black Africans) and those of other studies may be responsible. However, not much work has been done in this regard in Nigeria or Africa.

A study by Maas et al [1] has suggested a strong association between pregnancy, breast feeding and BAC. Pregnancy is associated with major changes in calcium metabolism to meet the high requirements for fetal growth and breast milk production. Pregnancy and breastfeeding induce transient hypercalcaemia. These may lead to calcium deposits in the breast arteries [1]. Contrary to the findings by Yildiz et al [6] that significantly linked increasing number of childbirths with BAC, there was no significant difference between the number of childbirths in subjects that had BAC and those that did not have BAC in this study. Our finding was also contrary to that of Maas et al [1].

The BMI was also not significantly higher in the subjects with BAC. This is contrary to the results from other studies [1-3]. We cannot explain the reason for this difference.

This study did not show any relationship between BAC and chronic diseases such as hypertension, and diabetes. This is in contrast with findings from studies by Kemmerem et al [2] and Oliveira et al [10].

## Conclusion

We report the prevalence rate of BAC to be 20% in our environment. Increasing age was the only factor which we found to be related to its presence. All the other CVD risk factors that were found in previous literatures were not associated with the presence of BAC in this study. Therefore, BAC found in screening and diagnostic mammography may not be a marker for CVD in women in our environment. To the best of our knowledge, not much work has been done in Nigeria and Africa on this. It is recommended that further studies should be done to elicit the reasons for the high prevalence and the differences in the risk factors among Nigerians.

## Competing interests

The authors declare that they have no competing interests.

## Authors' contributions

All authors have read and approved the final manuscript.

**RAA **Made substantial contribution to the conception and design of this study, acquisition and interpretation of data. She also helped in drafting and revising the script

**OAO **Contributed to data acquisition. Carried out the statistical analysis, point of care testing, sequence alignement and drafting of the script

**JAAO **Contributed to data acquisition. Carried out the laboratory analysis and coordinated the laboratory works of the subjects

**CEE **Contributed to data acquisition. Coordination of the clinical aspect of the study, taking the weights, Blood pressure, BMI, E.T.C

**IOF **He helped in interpretation of data, drafting, revising of the script
